# Case of Cleft Palate, Sarcomatous Testis, Double Genu-Valgus

**Published:** 1893-12

**Authors:** Thomas H. Manley

**Affiliations:** New York


ATLANTA

### MEDICAL AND SURGICAL JOURNAL.

VOL. X. DECEMBER, 1893. No. 10.

EDITED BY

LUTHER B. GRANDY, M. D., AND MILLER B. HUTCHINS, M. D.,

WITH THE CO-OPERATION OF

H. V. M. MILLER, M. D., LL. D., VIRGIL O. HARDON, M. D.,
FLOYD W. McRAE, M. D., AND ARTHUR G. HOBBS, M. D.

### *CLINICAL LECTURE.*

### CASE OF CLEFT PALATE, SARCOMATOUS TESTIS, DOUBLE GENU-VALGUS.*

BY THOMAS H. MANLEY, M. D.,

NEW YORK.

The baby about to be operated on for cleft palate, Dr. Manley said, was but seven weeks old; there was no history of maternal impression or heredity.

He said that in this class of cases the success attending operation was enhanced and danger minimized when they were taken in hand after the tenth year, as at this age the patient himself could assist very materially both in the operation and in the subsequent treatment. With them, at this age, local cocainization would suffice as an analgesic, so that the dangers of broncho-pneumonia following and the inspissation of blood would be obviated, and by sus-

*Clinic held at Harlem Hospital, New York, October 20, 1893.

pending oral alimentation for three or four days, until primary union was secured, the patient being amply nourished in the meantime by nutrient enemata. This was the stage of life at which the eminent Parisian surgeon, Trélat, recommended operative interference.

But in this case there are reasons why interference has been rendered imperatively early. The infant is unable to nurse the mother, and in swallowing a part of the fluid ingesta makes its way into the glottis and passes out through the nose.

As regards the technique of those uranoplastic operations, it must be acknowledged all operative procedures on the vault of the buccal cavity are attended with great manual difficulties, particularly during infantile life.

The operation would be begun by dividing the tensor and levator palati muscles; the vivifying the divided margins of the clefts and bringing them together with wire suture.

The second case, he said, was one of peculiar interest from a diagnostic point of view. The patient (a young man of twenty-six years) two months ago injured his right testis by a fall. Some sharp pain was felt in the gland for a week or ten days, but then wholly passed away. After about a month it reappeared, when he noticed an enlargement of the gland with an excoriation on the integument over the epididymis. This has since opened and discharged more or less surface serum and pus. Now, the question arises, what is the pathological basis of this ulcerating surface?

He has had constitutional syphilis six years ago. But this local condition is wanting in the clinical characters so common to this disease. In syphlitic orchitis or sarcocele the swelling is always painless, generally symmetrical and runs into ulceration. There are no reasons to suspect tubercle; besides the latter's most common seat is in the epididymis and runs into suppuration; while here the process is a hyperplasia and ulceration. The mass, however, has all the clinical characters of a malignant growth, aroused into activity by the injury. It is very painful, sensitive, is rapidly growing and taking on all the features of an encephaloid cancer.

Castration was performed, in the course of which it was observed that the growth has so infiltrated as to intimately involve the cord.

The next patient was a child of six years, who was suffering from double genu-valgus, or knock-knee of a rachitic origin. Dr. Manley said that he was induced to operate on this young, healthy child only at the urgent request of the parents, for the reasons: 1. That in the vast majority of cases the deflected shaft will straighten out before adult age is attained, and, further, that though an osteoclasis or osteotomy may bring the limb into an axis with the body, it will leave defective area at the point of fracture, which must result in a permanent weakening for the remainder of life.

